# Spatiotemporal, environmental, and behavioral predictors of *Varroa* mite intensity in managed honey bee apiaries

**DOI:** 10.1371/journal.pone.0325801

**Published:** 2025-08-07

**Authors:** Laura Boehm Vock, Lauren M. Mossman, Zoi Rapti, Adam G. Dolezal, Sara M. Clifton

**Affiliations:** 1 Department of Mathematics, Statistics, and Computer Science, St. Olaf College, Northfield, Minnesota, United States of America; 2 Department of Mathematics, University of California Davis, Davis, California, United States of America; 3 Department of Mathematics, University of Illinois at Urbana-Champaign, Urbana, Illinois, United States of America; 4 Department of Entomology, University of Illinois at Urbana-Champaign, Urbana, Illinois, United States of America; 5 Department of Mathematics, Denison University, Granville, Ohio, United States of America; University of Alberta, CANADA

## Abstract

Honey bees contribute substantially to the world economy through pollination services and honey production. In the U.S. alone, honey bee pollination is estimated to contribute at least $11 billion annually, primarily through the pollination of specialty crops. However, beekeepers lose about half of their hives every season due to disease, insecticides, and other environmental factors. Here, we explore and validate a spatiotemporal statistical model of *Varroa destructor* mite burden (in mites/300 bees) in managed honey bee colonies, exploring the impact of both environmental factors and beekeeper behaviors. We examine risk factors for *Varroa* infestation using apiary inspection data collected across the state of Illinois over 2018–2019, and we test the models using inspection data from 2020–2021. After accounting for spatial and temporal trends, we find that most environmental factors (e.g., floral quality, insecticide load) are not predictive of *Varroa* intensity, while lower numbers of nearby apiaries and several beekeeper behaviors (e.g., supplemental feeding and mite monitoring/treatment) are protective against *Varroa*. Interestingly, while monitoring *and* treating for *Varroa* is protective, treating *without* monitoring is no more effective than not treating at all. This is an important result supporting Integrated Pest Management (IPM) approaches.

## Introduction

Pollinators face substantial environmental challenges, often classified into four main stressors: parasites, pathogens, pesticides, and poor nutrition (the four P’s) [1]. Poor environmental conditions lead to weakened or even collapsed colonies, which has serious ecological and economic consequences [2–4]. An estimated 87.5% of flowering plants are pollinated by insects and other animals [5], and pollination services are estimated to contribute at least $235 billion annually in the world economy [6–8]. Honey bees (*Apis mellifera*) are of particular economic interest because the species is one of only a few pollinators domesticated for honey production and crop pollination [9].

Because of the importance of pollinators to the environment and economy, colony failure has attracted considerable attention from mathematical and statistical modelers. While the role of parasites and pathogens, such as *Varroa* mites and the viruses they vector (e.g., Deformed Wing Virus and Acute Bee Paralysis Virus) [10–12], remain the most studied culprits, other factors, such as pesticides [13] and poor nutrition [14], have also been considered. Every modeling effort has simplified the inherently complex pollinator system by focusing on a few aspects of the disease system while de-emphasizing other aspects.

For example, Kang *et al*. model bee-to-bee virus transmission explicitly by dividing the populations of both bees and mites into susceptible and infected [10]; seasonal effects, the role of nutrition, and mite dispersion among colonies are not considered. Other models focus attention on colony demography, seasonality, and food resources, while ignoring disease spread [14–16]. Still other models address disease and demographic dynamics, while ignoring environmental conditions [12, 17, 18].

While within-hive disease dynamics have been modeled with various levels of complexity [19], between-hive transmission over large spatial areas has been mostly neglected. This is understandable because large-scale colony parasite monitoring by single agencies, collecting and sharing data in a consistent manner, is sparse in space, time, and/or covariates. For example, the Bee Informed Partnership has performed the most widespread honey bee colony surveys over the last decade [20, 21]; however, these surveys focus primarily on annual and seasonal colony losses and do not usually incorporate more complex factors, practices, or exact location data. Other recent studies have modeled the transmission of various parasites and pathogens within a single apiary [22, 23], but to our knowledge very few modeling efforts (e.g., [24]) examine risk factors for parasites and pathogens (e.g., environmental conditions and beekeeper behaviors) over large spatial areas.

To fill the void on large spatial scale risk factors for *Varroa* infestation in managed honey bee colonies, we build and validate several spatiotemporal statistical models of *Varroa* intensity (mites/300 bees) in colonies across the state of Illinois (IL). To train and test the models, we merge several large-scale datasets of apiary inspections, beekeeper surveys, and environmental factors. After accounting for spatial and temporal trends (our baseline model), we compare the marginal effects of many factors hypothesized to increase risk for or protect against mite infestation. These factors include environmental conditions, such as floral quality, nesting quality, insecticide load, and apiary density. We also explore the impact of beekeeper interventions, including supplemental feeding, parasite monitoring, and various *Varroa* treatments.

## Methods

### Data

Our spatiotemporal data fall into three general categories: colony health status (with a location and a date), beekeeper behaviors (with a location and a year), and environmental indicators (with a location, sampled in 2018 but assumed to be stable over several years).

These data are collected from two main sources: (1) colony health status and beekeeper behaviors are available via yearly inspections in the state of Illinois from 2018 to 2021 [25], and (2) environmental indicators are available from Beescape (beescape.psu.edu). These sources cover all four of the main pollinator stressors (parasites, pathogens, pesticides, and poor nutrition) [1]. Because parasites and pathogens are highly correlated [26–29], we will use *Varroa* intensity as a proxy for both.

### Colony health status and beekeeper behaviors

The Illinois inspection data are thorough; each hive inspection includes the spatial location, multiple hive health indicators (e.g., queen status, amount of honey, egg/larvae presence), infestation status (e.g., *Varroa* mites per 300 bees, American or European foulbrood detection, small hive beetle presence), and disease control indicators (e.g., prophylactic treatments, beekeeper monitoring frequency and method, and intervention frequency and method). Beekeeper monitoring strategies include alcohol wash, sugar roll, drone cell examination, sticky board, and “other”. Reported treatment strategies include controlled brood break, fogging (wintergreen), oxalic acid (vapor or dribble), formic acid (vapor or pads), thymol, Amitraz, Hopguard, drone trapping, fluvalinate, and powdered sugar. Because monitoring and treatment details are not provided for most hives, we merge all monitoring strategies together, and we merge all treatment strategies together.

Although the parasite and pathogen data are thorough, they present several challenges and uncertainties. First, the inspection data are sparse in time; inspections of each hive occur only once per year, and only about one in five registered apiaries are inspected (it should be noted that this is a huge fraction of apiaries inspected relative to similar efforts, e.g., [20, 21]).

Second, while registration and inspections are mandated in Illinois, a non-negligible number of data points are missing important features (e.g., a valid location, date, or disease status); see Data Cleaning section for details.

Third, *Varroa* in particular must infest at least 1% of the hive to be detected; the standard method for testing is to remove and inspect 300 adult bees from a hive, with a limit of detection of one mite per sample (there is a correction factor of 2-3x because, for every one mite parasitizing adult bees, there are approximately two to three mites parasitizing pupae, which cannot be easily sampled) [30].

Finally, each data point is generated by a human inspector, and therefore may have errors or uncertainties that are not always easily quantified; see Data Cleaning section for details on removal or correction of human errors.

### Environmental factors

In addition to the inspection data, apiary registration data from the 2018 Colony Report, which includes latitude and longitude locations for all registered apiaries, was used to count the number of registered apiaries within a 5 km radius of our inspected sites, which we refer to as apiary density. Note that apiary density is not equivalent to any measure of colony density (e.g., bees per hive or hives within a certain radius).

Data relevant to insecticides and poor nutrition were scraped from the Beescape Map Tool [31] at each reported hive location (latitude and longitude) from 2018 inspections [32]. This application scores forage (floral) quality in spring, summer, and fall; nesting quality; and insecticide load [31] using a rigorously validated model for wild bee abundance [33, 34].

**Fig 1:**
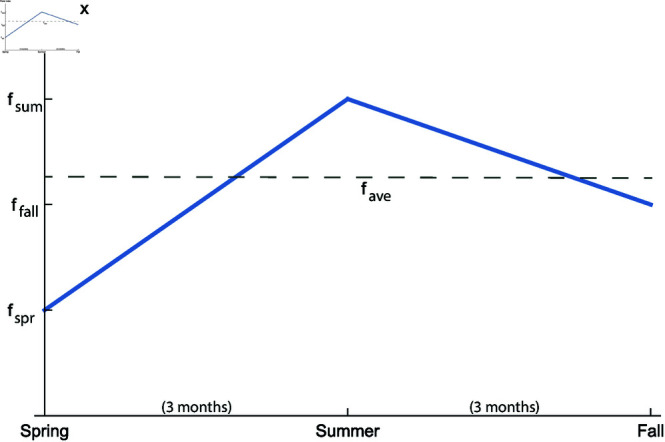
Our floral quality indicator is an estimated average of the seasonal floral indicators over the inspection period May through October. To estimate the average floral quality using the trapezoid rule, we assume spring to summer and summer to fall are three months each.

The forage quality score in each season is a weighted average of the density and supply of floral resources within a 3 km foraging radius of each hive location. The index, ranging from 0 to 100, is based on satellite imaging of natural areas and USDA crop surveys. Because each colony is inspected only once per year, sometime between May and October, and because all three seasonal floral indicators ($f_{spr},f_{sum},f_{fall}$) are highly correlated (from 0.91 to 0.99, depending on the pair) we use the estimated average floral quality over that six month period via the trapezoid rule assuming spring to summer and summer to fall are three months each (see Fig 1 for a visualization):


$$f_{ave}=\frac{1}{6}\left[\frac{3}{2}(f_{spr}+f_{sum})+\frac{3}{2}(f_{sum}+f_{fall})\right]$$

The nesting quality is estimated by averaging expert opinion on wild bee nesting quality provided by each land cover type. The nesting index, also ranging from 0 to 100, is not expected to be relevant for managed bees with man-made hives, and therefore serves as a check on our results.

The insecticide load score captures the expected toxic load of insecticides applied surrounding each reported hive location. The insecticide score is a weighted average of the lethal doses per area, scaled to fall in the same range as the nesting and forage indices. Theoretically, the insecticide score could take on any non-negative value, but the interquartile range is 79 to 226 across four representative U.S. states (Pennsylvania, Indiana, Illinois, and West Virginia).

**Fig 2:**
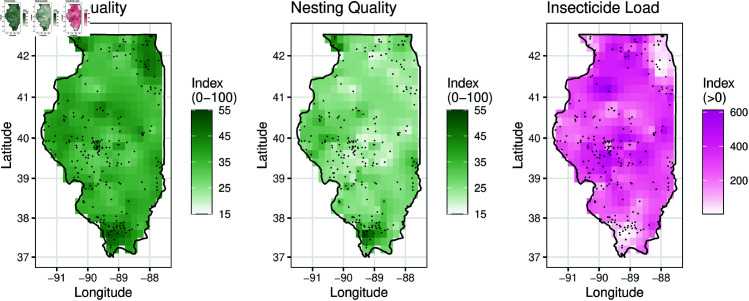
Floral quality, nesting quality, and insecticide load vary spatially over the state of Illinois. Environmental indicators are interpolated using kriging [35]. Black dots are apiary locations in 2018– 2021.

While we would have preferred to scrape the Beescape Map Tool each year to establish time-stamped environmental indicators at each inspected hive, the API became inaccessible after changing hosts around 2020. Therefore, we use the scraped environmental data from hive locations inspected in 2018 [25], and we spatially interpolate the indicators for locations inspected in subsequent years, assuming that the indicators remain relatively stable (see Fig 2).

### Data cleaning

The inspection data contain 1585 recorded inspections in the years 2018–2021. We remove all inspections without dates, which reduces the number to 1546. Of these, 1246 have valid latitude and longitude recorded. As latitude and longitude were manually entered, there are some mistakes such as missing minus signs or transposition of digits; we flag latitude and longitude as invalid if they mark locations outside the state of Illinois. For an additional 134 observations, we pull the latitude and longitude from the 2018 Colony Report by matching the registration ID.

The inspection data contain individual hive information. We combine observations at multiple hives from the same apiary (based on shared Latitude/Longitude) into a single value per apiary. If the *Varroa* intensity is measured at more than one hive at a single apiary, we calculate the average intensity value.

In total, this results in 266 unique apiaries in 2018–2019 which we use as training data for the models, and 140 apiaries in 2020–2021 which are used for model testing (see Tables 1 and 2 for summary statistics). The 266 apiaries in the training set had 216 unique beekeepers; due to limited duplication of ownership in the dataset, we did not account for individual beekeeper effects, only variables that describe beekeeping practices.

**Table 1:** Summary statistics for *Varroa* intensity in number of mites per 300 bees and potential environmental explanatory variables, Mean (SD). Note that the standard deviation for floral quality and insecticide load are lower for testing data than training data due to location variability. Training data (2018–2019) apiaries are located throughout the entire state. In 2020, there are hives near the Chicago area and in Central-Western IL only, and in 2021 only near Chicago. The testing data (2020–2021) do not include any apiaries in northwest or southern IL. Floral quality and insecticide load are more variable in southern IL in particular (see Fig 2), decreasing the SD for the testing data.

Variable	2018–2019 train	2020–2021 test
Total apiaries	N = 266	N = 140
Num. mites / 300 bees	4.7 (6.0)	4.9 (6.0)
Floral Quality	38 (6.5)	38 (3.1)
Insecticide Load	255 (160)	256 (65)
Number of Apiaries within 5 km	8.7 (9.2)	7.6 (8.7)

**Table 2:** Number of apiaries and summary statistics for *Varroa* intensity (number of mites per 300 bees) by monitoring/treatment regime and by supplemental feeding regime. N/A indicates response is missing in inspection report.

	2018–2019 (train)			2020–2021 (test)		
Variable	N	Mean (SD)	Median (Range)	N	Mean (SD)	Median (Range)
**Monitoring/Treatment Regime**						
Mon+Trt	149	3.96 (4.86)	2.5 (0–27.5)	53	3.32 (6.35)	1 (0–33)
Trt only	56	7.00 (8.03)	4.75 (0–37.5)	24	6.83 (5.89)	4 (0–19)
None	26	6.37 (7.52)	3.83 (0–36)	21	8.14 (4.99)	7.86 (0.5–20)
N/A	35	3.10 (3.5)	1.2 (0–11.5)	42	4.10 (5.24)	2 (0–20)
**Supplemental Feeding**						
Yes	211	4.35 (4.77)	2.75 (0–29.8)	113	4.82 (5.56)	3 (0–25)
No	53	6.30 (9.26)	3 (0–37.5)	27	5.12 (7.65)	3.5 (0—33)
N/A	2	2.00 (1.41)	2 (1–3)			

### Baseline model

As *Varroa* intensity is known to increase throughout the summer season [24, 36–38], we first consider a baseline model that incorporates this seasonal effect [39]. Additionally, our initial data exploration suggests that *Varroa* intensity also varies spatially. A Generalized Additive Model (GAM) allows us to flexibly model the nonlinear relationship between intensity and location [40]. Because intensity is measured as a count per 300 bees, we assume the response variable follows a Poisson distribution.

As not all intensities are reported as integers (due to both the averaging of multiple colony values per apiary, and some values being reported in fractions) and to account for the over-dispersion present in the data, we use a negative binomial model. Like the related Poisson regression model, a negative binomial model is suitable for count or rate data, but relaxes the Poisson assumption that the mean is equal to variance [41]. Instead, the variance of the distribution is related to the mean *μ* and the over-dispersion parameter *k*, such that $Var(Y)=\mu +\mu^{2}/k$.

The negative binomial regression model is written as

$$Y_{i}\sim \text{Neg. Binomial}(\mu_{i})\;\log(\mu_{i})=\beta_{0}+\beta_{1}{\text{Day}}_{i}+f(x_{i},y_{i}),$$ (1)

where *Y*_*i*_ is the *Varroa* intensity (averaged over all hives in the apiary) in mites/300 bees at each apiary *i*, $\mu_{i}$ is the mean (or expected) *Varroa* intensity, Day is the number of days since the beginning of the year (January 1 is Day 1) of the apiary inspection, and (*x*_*i*_, *y*_*i*_) are the longitude and latitude coordinates of the apiary. The relationship between Day and *log*(*Y*_*i*_) is assumed linear because exponential growth of *Varroa* intensity has been observed in previous studies [36], and a spline model is not significantly better than the linear model (*p* = 0.964). The function *f* is a low-rank thin plate regression spline, chosen because it has certain optimality properties for multidimensional (e.g., latitude and longitude) smoothing [42].

In our baseline model, we assume errors are not correlated spatially or temporally at a scale we can detect with our sparse data. After accounting for location and time of year, residuals are not spatially or temporally correlated, except possibly at very short spatial scales (<0.5 km); see Supplementary Materials for more details.

All models were fit using the mgcv package in R [43, 44] with smoothing parameters chosen via cross-validation.

### Stepwise regression

We first investigate the effect of beekeeper behavior and environmental variables, after accounting for location and time of year, by adding variables one at a time to the baseline model. We investigate both additive models, in which the effect of the variable is assumed to result in an increase in *Varroa* which is constant across time, as well as an interaction of each variable with day of year, which would result in different rates of growth of *Varroa* through the year.

Next, we investigate variables which were found statistically significant individually by adding them one at a time into a multiple linear regression model. As there were a small number (only treatment, supplemental feeding, and apiary density) we consider all three possible pairings and a fourth model which included all three variables.

Strong geographic trends in *Varroa* management practice lead to spatial confounding (and potential overfitting) in models which include both a spatial component and the management variable. We therefore repeat our analysis without the spatial component for comparison.

We evaluate model fit with AIC, percent deviance explained, relative error, and mean absolute error on the training data (2018–2019) and model prediction with relative error and mean absolute error on the test data (2020–2021).

AIC is the Akaike Information Criterion, which measures prediction error and is a relative measure of model quality which adjusts for the number of model parameters. The criterion is measured on a log scale, with lower values indicating better fit. Differences of >10 are considered large [45], while differences of <2 are considered negligible.

Percent deviance explained measures the reduction in model deviance in comparison to the null model. This is a metric of model fit similar to *R*^2^ but more appropriate for negative binomial regression. Higher values are better.

Relative error is


$$\frac{1}{N}\sum_{i=1}^{N}|Y_{i}-{\hat{Y}}_{i,\text{model}}|/{\hat{Y}}_{i,\text{base}}$$

Mean absolute error is


$$\frac{1}{N}\sum_{i=1}^{N}|Y_{i}-{\hat{Y}}_{i,\text{model}}|$$

where ${\hat{Y}}_{i,\text{model}}$ and ${\hat{Y}}_{i,\text{base}}$ indicated the predicted *Varroa* intensity from the specified model and the baseline model.

## Results

### Baseline model

*Varroa* intensity is known to vary over space and time, regardless of beekeeper behaviors or environmental factors [24, 37, 38]. Therefore, we fit and validate a baseline model that incorporates these spatiotemporal effects [39] in order to understand the marginal impact of other factors. Like other observational studies, our baseline model confirms an exponential increase in *Varroa* intensity over time [36]. As expected, we also find substantial spatial differences in *Varroa* intensity across Illinois (hives are spread across a wide variety of landscapes, from high-intensity agricultural areas to dense urban areas like Chicago).

**Fig 3:**
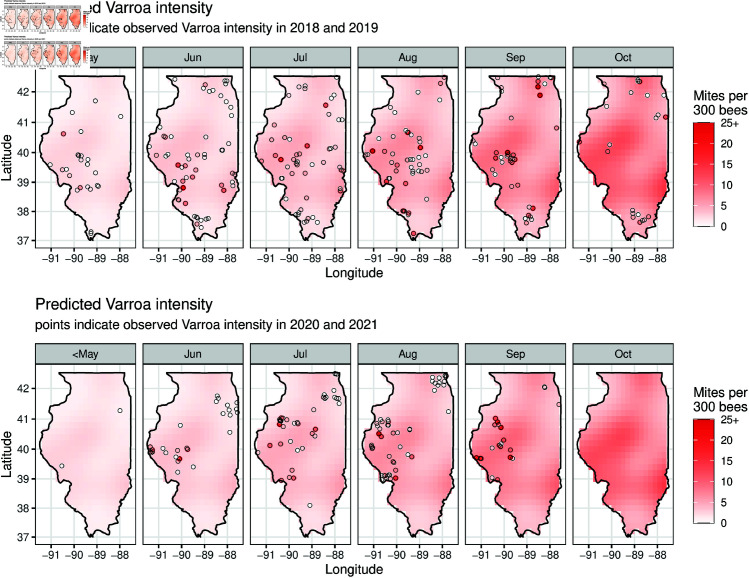
Predicted Varroa intensity varies geographically, with patterns consistent from training to test data. Red gradient indicates *Varroa* intensity predicted from baseline model (i.e., the model using only day of year and spatial location) using 2018–2019 data. Predictions are for the first day of indicated month. Top row: predicted intensity with observed 2018–2019 intensities as overlaid dots (training). Bottom row: predicted intensity with observed 2020–2021 intensities as overlaid dots (testing).

**Fig 4:**
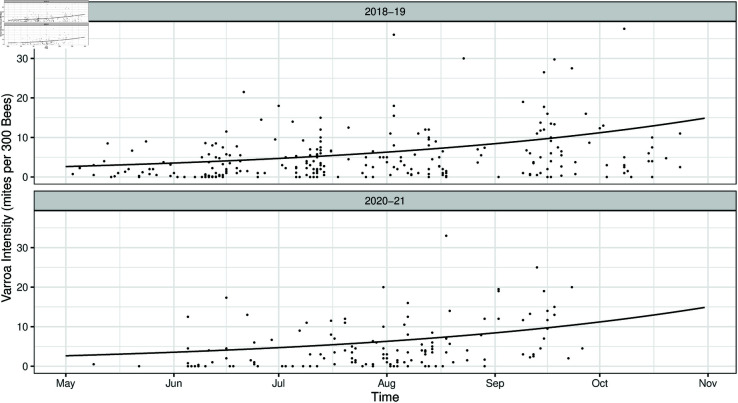
Varroa intensity increases through the summer months. *Varroa* intensity predicted from baseline model at the center of the state of Illinois (–89.68^*o*^, 39.90^*o*^) using 2018–2019 data (solid line). Dots indicate observed *Varroa* intensities. Top row: predicted intensity with observed 2018–2019 intensities overlaid (training). Bottom row: predicted intensity with observed 2020–2021 intensities overlaid (testing).

In Fig 3 we see the predictions from the baseline model in each month over the entire state of Illinois. The predicted surface is fit with the 2018–2019 training data on the first day of each month. We can compare the observed *Varroa* intensities in the training data (2018–2019) and in the test data (2020–2021) to the surface predicted by our model. To see the smooth trend in time, we predict the *Varroa* intensity at the center of the state (-89.68^*o*^ longitude, 39.90^*o*^ latitude) each day from May 1 to October 31 (Fig 4).

### Impact of environmental factors

Environmental variables, like local nutrition quality and insecticide burden, are expected to influence *Varroa* intensity because of several complex interactions between bee health, collective behavior and parasite dispersal [2–4]. The environmental variables tested are floral quality, insecticide burden, nesting quality, and apiary density; surprisingly, only the number of nearby apiaries appears to have a marginal impact on *Varroa* intensity.

Floral quality, insecticide use, and nest quality are not significant as shown in Table 3. While the additive model for apiary density, measured as the number of apiaries within a 5 km radius, does not show statistical significance, the model which includes an interaction between day of year and apiary density is a significant improvement over the baseline model. This suggests that the growth rate for *Varroa* increases as the number of nearby apiaries increases; however the estimated initial *Varroa* intensity at May 1st is lower in regions with higher apiary density. Note that in our sample, there does not seem to be an association between treatment strategy and apiary density, either globally or regionally. The predicted level of *Varroa* is lower at high density regions until mid August, and then is higher than in low density regions. Graphical results indicate this relationship could be driven by influential late season observations in very high density regions (see Supplemental Materials). The model with apiary density and its interaction with time has the lowest relative error and mean absolute error, both for the training and testing data, indicating that apiary density is important for *Varroa* intensity prediction, particularly late in the season.

**Table 3:** Comparison of models predicting *Varroa* intensity, with spatial component. For each variable, we fit an additive model and interaction with time; the model preferred by AIC is presented here. This table includes the results of the additive model for Floral quality, Insecticide, and Nesting quality. The remaining models include the indicated variables and interaction with time. Note that management practices are correlated with location, leading to possible overfitting in models that include the management variable. Statistics presentedeak are: Akaike Information Criterion (AIC), % of Deviance explained (a metric similar to $R^{2}$ but more appropriate for negative binomial regression), Relative Error (RelErr = Mean($|y\;-\;{\hat{y}}_{model}|/{\hat{y}}_{base}$), Mean Absolute Erroreak (MAE = Mean($\left|y\;-\;{\hat{y}}_{model}\right|$)). The null model uses overall mean *Varroa* intensity as the predicted value ($\hat{y}$). The best model for each statistic is bolded. There is no clear winner among the models for all criteria.

	Training: 2018–2019				Test: 2020–2021	
Model	AIC	% Dev. Expl.	Rel. Err.	MAE	Rel. Err.	MAE
Null	1244	0.00	1.05	4.18	1.03	4.57
Baseline (Space, Time)	1222	0.17	0.80	3.66	0.80	3.87
**Environmental variables**						
Floral Quality	1224	0.17	0.79	3.65	0.80	3.86
Insecticide	1224	0.17	0.79	3.65	0.80	3.86
Nesting quality	1222	0.17	0.80	3.66	0.80	3.86
Apiary Density	1219	0.19	**0.77**	**3.57**	**0.77**	**3.80**
**Beekeeper variables**						
Management	1220	0.16	0.83	3.90	0.80	3.93
Supp. Feed	**1197**	0.24	0.81	3.73	0.85	4.62
**Multiple regression**						
Dens/Manage	1220	0.19	0.80	3.80	0.79	3.90
Dens/Supp	**1197**	**0.25**	0.79	3.65	0.84	4.54
Manage/Supp	1201	0.24	0.80	3.79	0.81	4.43
Dens/Man/Supp	1201	**0.25**	0.78	3.70	0.80	4.39

### Impact of beekeeper interventions

While seasonality, geographical location, and environmental conditions are largely outside of beekeeper control, the beekeeper selects apiary management practices. Therefore, we explore the marginal impact of beekeeper interventions on *Varroa* intensity, after accounting for spatiotemporal effects. The beekeeper intervention variables tested are supplemental nutrition (syrup, pollen, and solids) and parasite management (various monitoring and treatment strategies, as outlined in the Data section). We find that supplemental feeding and parasite monitoring with treatment appears to slow *Varroa* growth, but treating for parasites without monitoring has no marginal effect.

The majority of the managed apiaries in our dataset (214, or 80%) provide supplemental feeding. Of these, the most common supplement is syrup only (135), or syrup in combination with pollen (29), solid (17), or both pollen and solid (15). Only 15 total apiaries provided supplemental feeding of solids, pollen, or combination, without syrup.

The practice of supplemental feeding does not seem to vary across the state; the predominant practice (80% of apiaries observed in 2018–2021) across all regions is to provide supplemental feeding; this makes sense as feeding is widely recommended at some points of the year and is therefore a typical beekeeping practice [46–48]. Comparison of the additive and interaction models indicate that the interaction model which allows for growth in *Varroa* intensity to depend on supplemental feeding practice is a significant improvement. The rate of growth of *Varroa* intensity is 2.5% per day in apiaries that do not supplement, and only 0.9% per day in apiaries that supplement (*p*-value for test of difference is 0.0001). The model with supplemental feeding and time interaction has the lowest AIC of all single variable regression models, indicating good fit to the training data. It does not however improve model prediction as well as apiary density, perhaps because supplemental feeding is so common.

Among many possible *Varroa* management strategies, we consider three categories: “Monitor + Treat", “Treat only" and “None", with the monitor and treat strategy being predominant, as seen in Table 2. Due to small sample size, we did not include a “Monitor only” category. There were only 10 apiaries which reported monitoring, but not treatment in 2018–2019. Of these, three had responses to other variables which indicated they may possibly treat (e.g. a last treatment date of 2017, reported treatment frequency of >0 in last 12 months). Therefore these 10 apiaries are included in the “Monitor + Treat” category. As a sensitivity analysis, we repeated the analysis with these 10 apiaries in the “None” (no treatment) category, and achieved similar results. Integrated Pest Management (IPM) approaches recommend only treating in conjunction with testing, in order to reduce ecological contamination and pesticide resistance [49]; therefore, any beekeepers treating without testing are not following IPM best practices.

The interaction model shows that the growth rate of *Varroa* over time is significantly higher for the “Treatment only” group compared to the “Monitor + Treatment” strategy (*p*-value = 0.0034). The growth rate for “Monitor + Treat” is 0.5% per day, but for “Treatment only” is 1.7% per day. The “No treatment” group is not significantly different from either of the other groups, although this may be at least partially due to small sample size in that group. Because the sample size on the “no treatment” group is small, the uncertainty in the estimated effect is quite large (it has large standard error). The growth rate for the “No treatment” group is also estimated in between the other two groups (about 1% per day). Since these differences are smaller, and the standard error is larger, the “No treatment” group is not statistically significantly different from either “Treatment only” or “Monitor + Treat.”

Though we observe significant differences between management strategies, we see that the AIC is only slightly lower for the management model (1220) compared to baseline (1222) (Table 3). Despite adding terms to the linear part of the model, the spatial component of the model is reduced in complexity such that the total model degrees of freedom is lower in the model that include management strategies than the baseline. This suggests management is an important variable, though highly confounded with location. For example, in central Illinois, where predicted *Varroa* intensity is high, there are a variety of monitoring and treating practices. In Southern Illinois, where predicted intensity is low, nearly everyone monitors. It is not clear if these geographic trends in common practices reflect a response to local conditions or the sharing of practices and knowledge among local beekeepers.

### Multiple regression model

Based on our stepwise regression results, we have identified three variables which are individually important for predicting *Varroa* intensity: apiary density, *Varroa* management strategy, and supplemental feeding practice. We test each combination of these variables to find the best model. Because we observe that the impacts of management strategy are confounded by spatial location, we compare the 7 potential models with (Table 3) and without (Table 4) the spatial component. There is no clear winner among the models for all criteria. When the spatial component of the model is included (Table 3), including management strategy does not lead to improvements in model fit after accounting for the number of apiaries within 5 km (apiary density) or supplemental feeding. However, if the spatial component is excluded (Table 4), management strategy is important, particularly when considered in conjunction with supplemental feeding. Because of the spatial patterns in management strategy mentioned in the previous section, it is difficult to disentangle these effects. We do see however, that apiary density is important after accounting for this spatial heterogeneity either through inclusion of the spatial component (Table 3) or management strategies (Table 4), both in improving the model fit for the 2018–2019 data, and in predicting *Varroa* intensity in 2020–2021. After accounting for apiary density and location (and/or management strategy), supplemental feeding may improve the 2018–2019 model fit (as measured by AIC), but does not improve prediction for 2020–2021.

**Table 4:** Comparison of models predicting *Varroa* intensity, without spatial component. For each variable, we fit an additive model and interaction with time; the model preferred by AIC is presented here. This table includes the results of the additive model for Floral quality, Insecticide, and Nesting quality. The remaining models include the indicated variables and interaction with time. Statistics presented are: Akaike Information Criterion (AIC), % of Deviance explained (a metric similar to $R^{2}$ but more appropriate for negative binomial regression), Relative Error (RelErr = Mean($|y\;-\;{\hat{y}}_{model}|/{\hat{y}}_{base}$), Mean Absolute Error (MAE = Mean($\left|y\;-\;{\hat{y}}_{model}\right|$)). Relative error is computed relative to the baseline non-spatial model (time only). The best model for each statistic is bolded. There is no clear winner among the models for all criteria, however all the highlighted models include Management as a variable.

	Training: 2018–2019				Test: 2020–2021	
Model	AIC	%Dev. Expl.	Rel. Err.	MAE	Rel. Err.	MAE
Time (base)	1223	0.08	0.83	3.98	0.88	4.23
**Environmental variables**						
Floral quality	1224	0.09	0.83	3.97	0.88	4.19
Insecticide	1223	0.09	0.82	3.95	0.87	4.18
Nesting quality	1225	0.08	0.83	3.98	0.88	4.23
Apiary Density	1222	0.10	0.82	3.91	0.87	4.18
**Beekeeper variables**						
Management	1214	0.14	0.81	3.95	0.86	4.05
Supp. Feed	1210	0.14	0.83	3.86	0.95	4.58
**Multiple regression**						
Dens/Manage	1215	0.15	**0.79**	3.88	**0.85**	**4.03**
Dens/Supp	1210	0.15	0.81	**3.82**	0.93	4.50
Manage/Supp	**1205**	0.18	0.80	3.84	0.90	4.29
Dens/Man/Supp	1206	**0.19**	**0.79**	**3.82**	0.89	4.25

**Fig 5:**
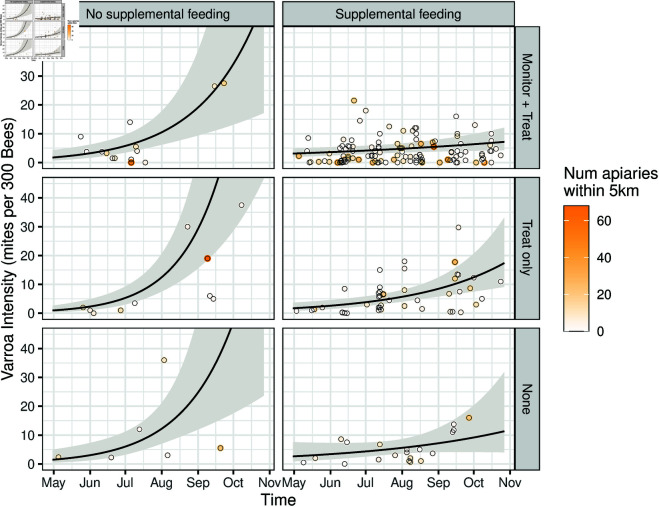
Supplemental feeding and monitoring and treating for Varroa both may reduce intensity and growth rate through the summer. Observed *Varroa* intensity (points) and predicted intensity (line) from multiple regression model without spatial component using 2018-2019 data, by supplemental feeding and management practice categories. Line indicates predicted mean *Varroa* (gray band = 95% confidence interval) predicted when number of apiaries within a 5 km radius is 5 (the median value).

Fig 5 and Table 5 show the predicted *Varroa* intensity over the course of a season and the predicted daily growth rate from the multiple regression model which includes Supplemental Feeding (Yes/No), *Varroa* management practice (Monitor + Treat, Treat only, None) and apiary density (number of apiaries within 5 km). Table 6 shows the positive relationship between apiary density and growth rates in the “Monitor + Treat” with “Supplmental feeding” group.

**Table 5:** Predicted *Varroa* intensity and daily *Varroa* growth rate from multiple regression model with spatial component. Values are estimated for an apiary density of 5 apiaries within 5 km (the median value). May 1 intensity is the mean *Varroa* intensity (mites per 300 bees) predicted for May 1; Growth rate is the daily growth rate of *Varroa* as a multiplicative factor; Growth % is the daily growth rate of *Varroa* as percent change (0–100).

Management	Feeding	May 1 intensity	Growth rate	Growth %
Monitor + Treat	No supp. feeding	1.74	1.020	1.95
Monitor + Treat	Supp. feeding	3.12	1.005	0.46
Treat only	No supp. feeding	0.97	1.028	2.79
Treat only	Supp. feeding	1.73	1.013	1.29
None	No supp. feeding	1.46	1.023	2.31
None	Supp. feeding	2.62	1.008	0.82

**Table 6:** Predicted *Varroa* intensity and daily *Varroa* growth rate from multiple regression model (with spatial component) assuming “Monitor + Treat” management strategy and “Supplemental feeding.” Values are estimated for varying apiary density, from 0 to 60 apiaries within a 5 km radius. May 1 intensity is the mean *Varroa* intensity (mites per 300 bees) predicted for May 1; Growth rate is the daily growth rate of *Varroa* as a multiplicative factor; Growth % is the daily growth rate of *Varroa* as percent change (0–100).

Apiary density	May 1 intensity	Growth rate	Growth %
0	2.13	1.011	1.12
1	2.04	1.012	1.16
2	1.96	1.012	1.19
5	1.73	1.013	1.29
10	1.40	1.015	1.46
20	0.93	1.018	1.80
60	0.17	1.032	3.16

The sample sizes for the “No supplemental feeding” groups are small, leading to large uncertainty bounds; however, the predicted *Varroa* intensity and growth rate are statistically significantly higher (*p*-value = 0.003) than for the “Supplemental feeding” group. Supplemental feeding seems to have the largest effect size. There is not a significant difference (*p*-value = 0.484) between the “Monitor + Treat” and “None” management groups, but the “Treat only” group has significantly higher growth rate than “Monitor + Treat” (*p*-value = 0.031). This suggests supplemental feeding along with a *Varroa* monitoring and treatment regime may be the best strategy to keep *Varroa* intensity low throughout the season.

## Discussion

With the goal of understanding the main predictors of *Varroa* infestation in managed apiaries over large spatial areas, we train and test several spatiotemporal models using four years of apiary inspection data in the state of Illinois. Like many ecological datasets, our data have limited resolution in both space and time; this makes validation of mechanistic models challenging. Therefore, we use statistical models to probe the risk factors for *Varroa*, including time of year, location, apiary density, several environmental factors, and several beekeeper behaviors.

Our baseline model, accounting for only time of year and location shows that *Varroa* intensity grows exponentially in time, as shown in other studies (e.g., [36]), and is also spatially varying. After accounting for location and time of year, nesting quality for wild bees is not predictive of *Varroa* intensity, as expected; our dataset is comprised entirely of managed apiaries. However, it is surprising that other environmental factors such as floral quality and insecticide burden are also not predictive of *Varroa* intensity. Other studies show that limited environmental nutrition exacerbates parasite load [50–53]. That said, the widespread practice of supplemental feeding in our dataset may nullify the effects of poor nutrition in the natural environment.

Although high insecticide loads have been associated with weakened immune response of bees [2], we do not find an association with *Varroa* load. Our work aligns with other observational studies that show *Varroa* presence itself erases the influence of any environmental factors [4]; however, in a controlled experiment, it has been found that insecticide exposure increases *Varroa* intensity [54]. It is possible that our study does not sufficiently capture the pesticide burden in the area, as Beescape’s insecticide index ignores sublethal insecticide levels and excludes all fungicides and herbicides.

After accounting for spatiotemporal effects, increased apiary density appears to be a risk factor for infestation. Note that apiary density (number of apiaries within 5 km radius) and colony density (either number of colonies within a certain radius, or bees per hive) are not interchangeable measurements of density. To our knowledge, apiary density has not been studied as a risk factor for *Varroa*. Studies show that bees engaged in robbing are more likely to be infected with pathogens [55–59]. Late in the year, failing or weak colonies are often robbed by healthy colonies, which could contribute to greater *Varroa* transmission in areas with higher apiary density [56, 60].

After accounting for location and time of year, certain beekeeper behaviors (e.g., supplemental feeding, monitoring and treating for *Varroa*) appear to significantly reduce the *Varroa* growth rate, while treating without monitoring appears to offer no benefit over no treatment. This confirms other studies showing that supplemental nutrition and monitoring reduce parasite load [61]. It is also a logical result; if beekeepers are treating only, they rely on guesswork and luck to treat at the right time for efficacy. That said, some beekeeper behaviors (e.g., monitoring for *Varroa*) are correlated with location. Therefore, it is challenging to know if location itself or beekeeper monitoring is a predictor of infestation. Measurable impacts of treating and monitoring may also be affected by the variety of treatment methods and timing used, as well as other mitigating factors like using *Varroa*-resistant bee stocks.

### Limitations

While ideally a study like this would disambiguate the role of location/time, environmental conditions and beekeeper behaviors in exacerbating or protecting against *Varroa* infestation, sparse and missing data limit our conclusions. Comprehensive, consistent, and time-dense data collected over large spatial areas are not currently available for honey bee colonies. Arguably the most comprehensive data collector is the Bee Informed Partnership (beeinformed.org), but the data are not publicly available, and the organization is reducing its operations.

Another spatial complexity for which we do not account is the common practice of migratory beekeeping, which has also been linked to pollinator disease spread [62–64]. It is speculated that bees shipped thousands of miles to satisfy seasonal pollination demand will experience an increased risk of parasitism and infectious disease compared with colonies that remain in a single location, although rigorous experimental studies have not been performed to verify the observational claim [65]. Parasite and pathogen spread through migratory beekeeping remains an understudied phenomenon, despite the practice’s frequency and importance in agriculture [65].

Additionally, our study uses data from the state of Illinois only. No study of honey bees will be completely free of locality, as honey bees span most of the globe and experience many different environments. Here, we use a comprehensive dataset in a region that contains a gradient from urban-rural and includes some of the most extensively farmed landscapes in the world. More study is needed to understand whether the patterns observed here are generalizable outside of a region like Illinois.

Finally, correlations (even where our evidence is significant) do not imply causation. The existence or direction of causal relationships among time/location, environmental conditions, beekeeper behaviors, and parasite load remain less clear, primarily because causal studies are difficult and expensive to perform across large spatial scales. Both dense ecological data and controlled experiments are needed to resolve these ambiguities. Smaller-scale causal studies remain necessary to understand the relationships among disease burden, environmental conditions, and beekeeper interventions.

## Conclusion

Using a spatiotemporal model of *Varroa* infestation in managed apiaries across the state of Illinois over four years, we test the correlations among parasite intensity, environmental conditions, and beekeeper behaviors. Surprisingly, we find that environmental factors, such as floral quality and insecticide burden, are not predictive of *Varroa* growth. On the other hand, factors largely within beekeeper control (e.g., supplemental feeding and parasite monitoring/treatment) predict mite intensity. While not under a single beekeeper’s control, apiary density also predicts mite intensity. Smaller apiary density and supplemental feeding appear to be protective against *Varroa* growth. Interestingly, while monitoring *and* treating for mites is protective, treating *without* monitoring is no more effective than not treating at all. This is an important result supporting Integrated Pest Management (IPM) approaches.

## Supporting information

### Investigating spatial and temporal autocorrelation

After fitting the baseline negative binomial regression model (Eqs 1), we examine the residuals for spatial and temporal autocorrelation.

The empirical semivariogram (S1 Fig) indicates no detectible spatial autocorrelation even at the smallest lag distance (<1 km), though it is possible spatial correlation could exist at a finer scale. Additionally, spatial correlation may not be detectable because measurements that were taken close together spatially were not necessarily taken close together in time.

S1 Fig Empirical semivariogram of residuals from baseline model using 2018–2019 training data. Bin width is 1 km. (TIF)

We also examine the temporal autocorrelation for both the residuals and for the measured *Varroa* intensity (S2 Fig), which shows no detectable autocorrelation. However, this result only demonstrates that we are unable to detect autocorrelation with the data available, not that no autocorrelation exists. Of the 540 available days of data from the two seasons of the training data, *Varroa* intensity is only measured on 134 unique days, and only 61 pairs of consecutive days. Additionally, even if measurements are available on consecutive days, they may be far apart in space, thus making the temporal autocorrelation difficult to detect.

S2 Fig Empirical lagged autocorrelation function (ACF). (TIF)

### Possible interaction between apiary density and time to predict *Varroa* intensity

As described in the section “Impact of environmental factors”, apiary density is not statistically significant in a single variable additive model, but the interaction between apiary density and time of year does appear significant. S3 Fig and S4 Fig show that this association may be driven by high late-season *Varroa* intensity for apiaries with very high nearby apiary density. Predicted intensity is similarly low for all values of apiary density until mid-August, when the predicted intensity for the higher density regions increases rapidly.

S3 Fig Varroa intensity over time for different levels of apiary density. Each point represents one apiary, panel labels indicate approximate number of colonies within a 5 km radius. Black line indicates the *Varroa* intensity in mites per 300 bees as predicted from a model with interaction between day of year and apiary density, along with gray 95% confidence band.
(TIF)

S4 Fig Predicted *Varroa* intensity from model with interaction of apiary density and day of year. These are the same models shown in S3 Fig.
(TIF)
