# Supplementary figures and images for: Spatiotemporal, environmental, and behavioral predictors of Varroa mite intensity in managed honey bee apiaries
Source: PLoS One. 2025 Aug 7;20(8):e0325801. doi: 10.1371/journal.pone.0325801 (PMC12331053; doi:10.1371/journal.pone.0325801)

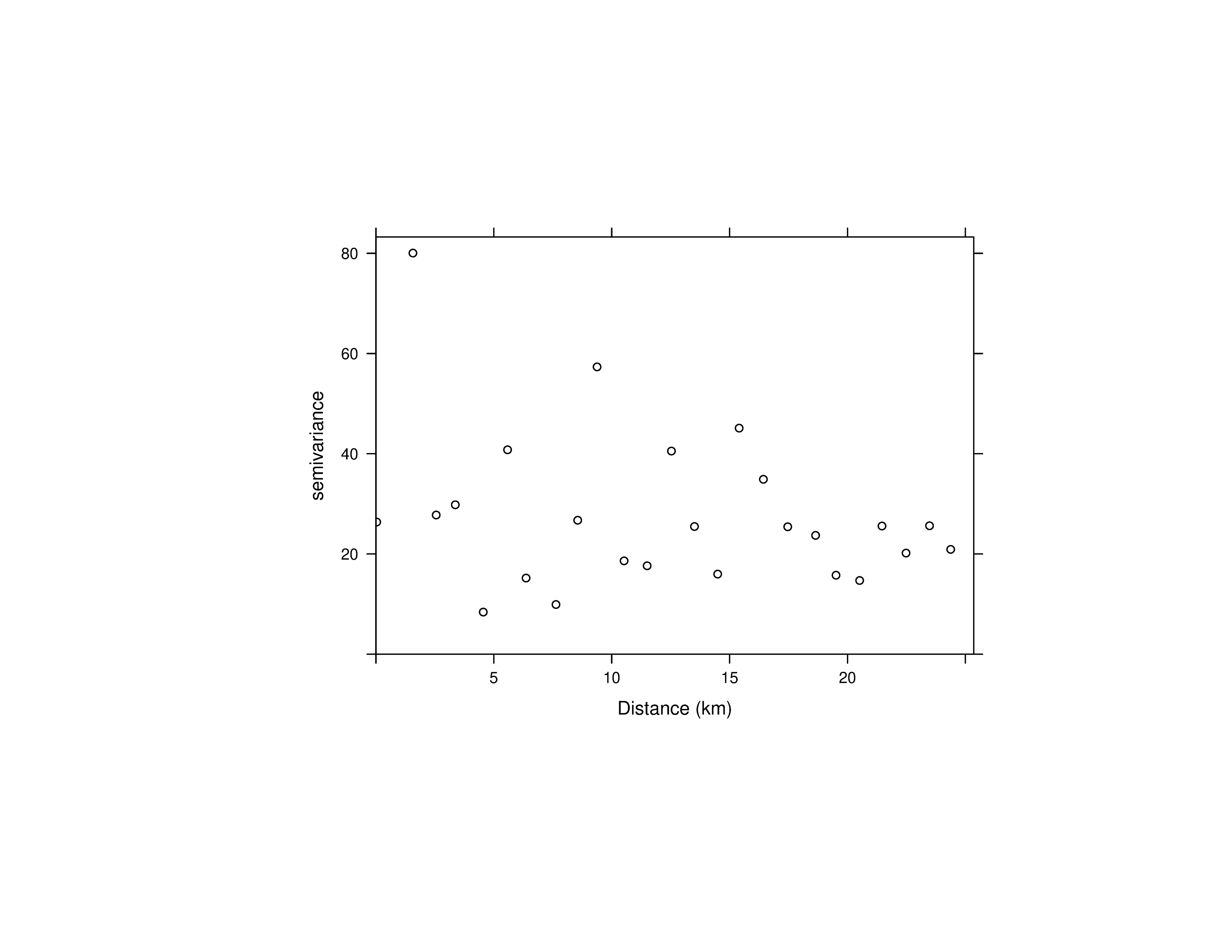

Supplement: S1 Fig — (TIF) [file pone.0325801.s001.tif]

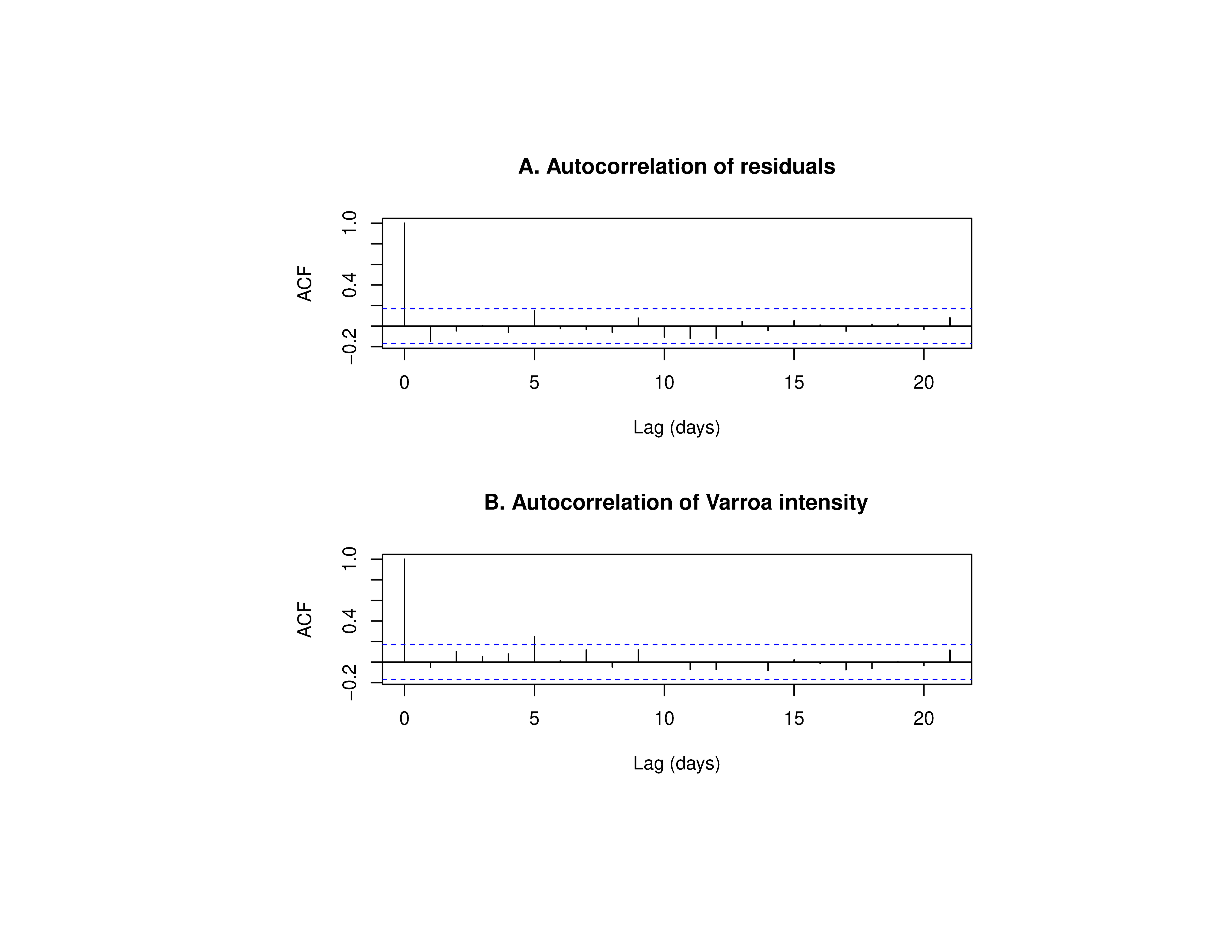

Supplement: S2 Fig — (TIF) [file pone.0325801.s002.tif]

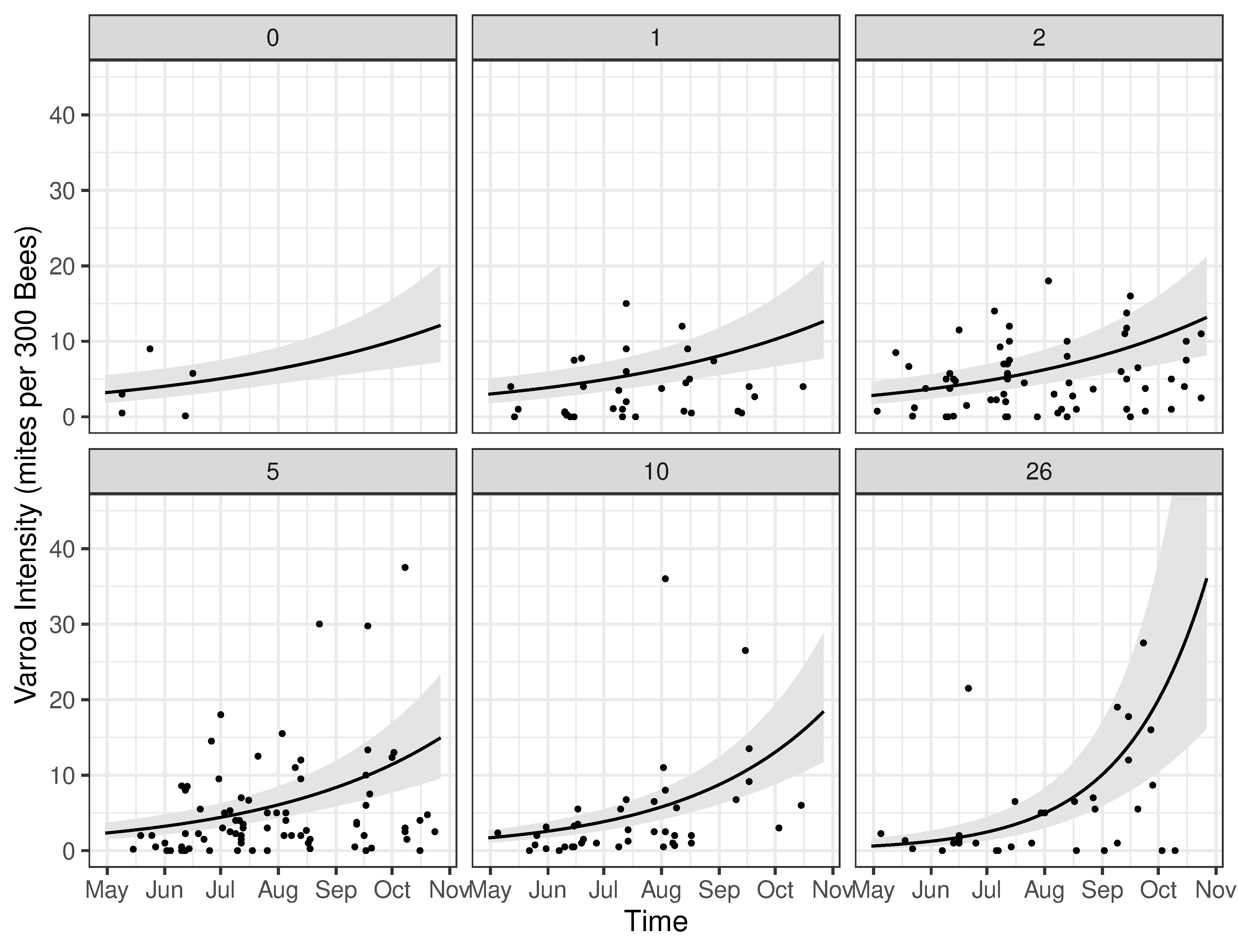

Supplement: S3 Fig — Each point represents one apiary, panel labels indicate approximate number of colonies within a 5 km radius. Black line indicates the Varroa intensity in mites per 300 bees as predicted from a model with interaction between day of year and apiary density, along with gray 95% confidence band. (TIF) [file pone.0325801.s003.tif]

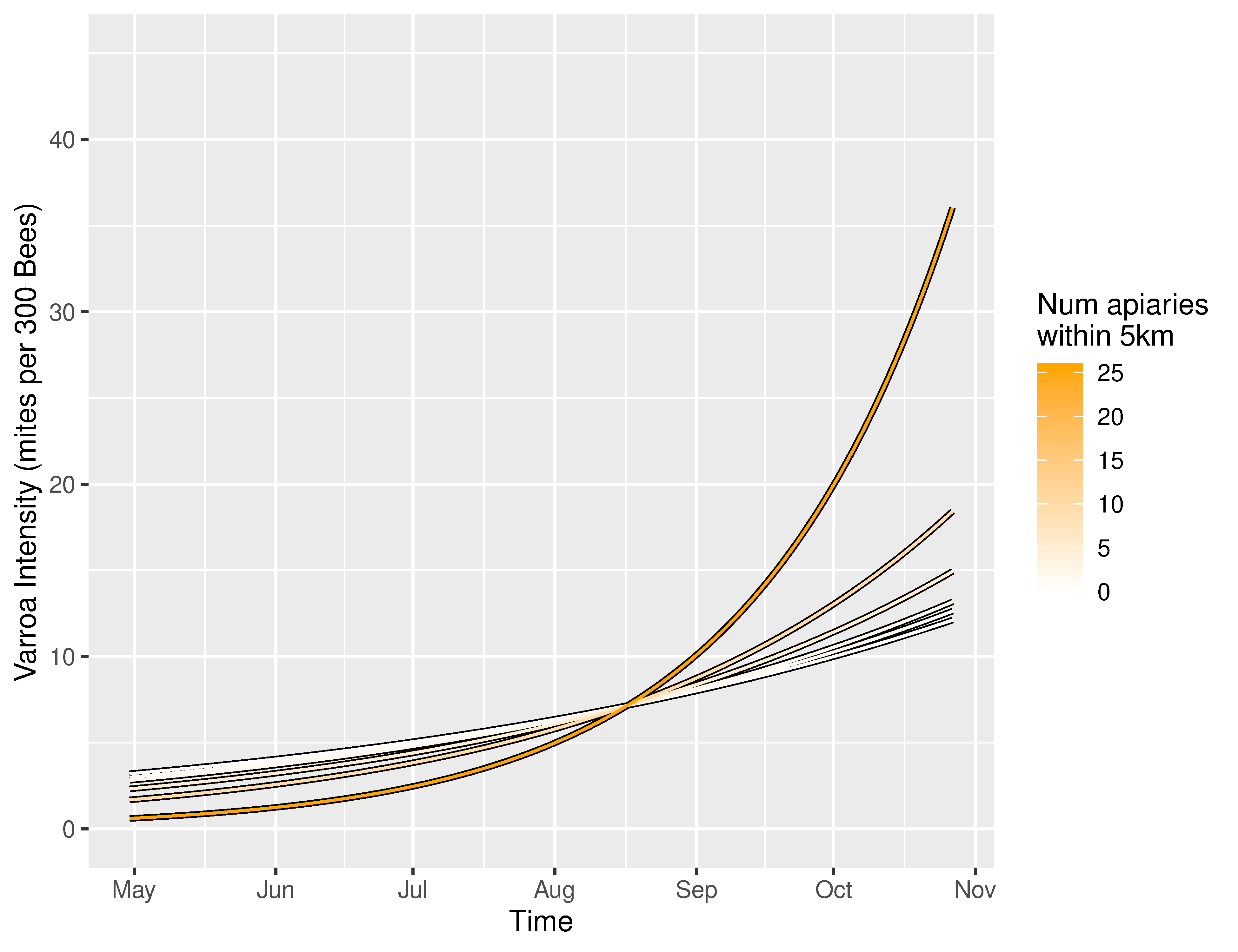

Supplement: S4 Fig — These are the same models shown in S3 Fig. (TIF) [file pone.0325801.s004.tif]
